# The Second Heart Program—A multidisciplinary team supporting people who inject drugs with infective endocarditis: Protocol of a feasibility study

**DOI:** 10.1371/journal.pone.0256839

**Published:** 2021-10-28

**Authors:** Robin Lennox, Larkin Lamarche, Leslie Martin, Tim O’Shea, Emilie Belley-Côté, Anna Cvetkovic, Olivia Virag, Richard Whitlock

**Affiliations:** 1 Department of Family Medicine, McMaster University, Hamilton, Ontario, Canada; 2 Department of Medicine, McMaster University, Hamilton, Ontario, Canada; 3 Division of Infectious Diseases, Department of Medicine, McMaster University, Hamilton, Ontario, Canada; 4 Division of Cardiology, Department of Medicine, McMaster University, Hamilton, Ontario, Canada; 5 Population Health Research Institute, Hamilton, Canada, Hamilton, Ontario, Canada; 6 Division of Cardiac Surgery, Department of Surgery, McMaster University, Hamilton, Ontario, Canada; PLOS, UNITED KINGDOM

## Abstract

**Introduction:**

Infective endocarditis (IE) is a severe and highly prevalent infection among people who inject drugs (PWID). While short-term (30-day) outcomes are similar between PWID and non-PWID, the long-term outcomes among PWID after IE are poor, with 1-year mortality rates in excess of 25%. Novel clinical interventions are needed to address the unique needs of PWID with IE, including increasing access to substance use treatment and addressing structural barriers and social determinants of health.

**Methods and analysis:**

PWID with IE will be connected to a multidisciplinary team that will transition with them from hospital to the community. The six components of the Second Heart Team are: (1) peer support worker with lived experience, (2) systems navigator, (3) addiction medicine physician, (4) primary care physician, (5) infectious diseases specialist, (6) cardiovascular surgeon. A convergent mixed-methods study design will be used to test the feasibility of this intervention. We will concurrently collect quantitative and qualitative data and ‘mix’ at the interpretation stage of the study to answer our research questions.

**Ethics and dissemination:**

This study has been approved by the Hamilton Integrated Research Ethics Board (Project No. 7012). Results will be presented at national and international conferences and submitted for publication in a scientific journal.

**Clinical trail registrarion:**

Trial registration number: ISRCTN14968657 https://www.isrctn.com/ISRCTN14968657.

## Introduction

Within Canada, the number of persons who inject drugs (PWID) has been increasing, with an estimated 171,900 individuals or 0.7% of the population identified as PWID in 2016 [1]. Infective endocarditis (IE) is a severe infection among PWID associated with an increasing incidence rate in Ontario, Canada, since 2010 [2]. PWID have similar short-term (30-day) outcomes following both medically-managed and surgically-managed IE to non-PWID [3,4]. However, their long-term mortality rates after IE are high: 26% at 1-year, 42% at 5-years, and 56% at 10-years [4]. Common causes of death include reinfection with IE [4] and sepsis [5].

Despite the well-established link between ongoing substance use and the risk of complications following IE, very little is done in hospital to address underlying substance use disorders among PWID with IE. A retrospective review of 102 patients hospitalized with injection drug use-associated IE (IDU-IE) in Boston found that only 23.7% of those patients received an addiction consultation while hospitalized and only 7.8% of those had a plan for medication-assisted treatment, such as opioid agonist therapy, upon discharge [6]. Expanding access to substance use treatment could yield substantial benefit: in one study, receiving opioid agonist therapy was associated with 70% reduction in mortality following IDU-IE [7]. The current lack of intervention to reduce the harm of patients’ substance use in the context of their IE likely contributes to the high rates of reinfection, re-intervention, and the disproportionate mortality rates experienced by PWID following discharge from hospital.

PWID also have many non-medical risk factors that likely impact their long-term IE outcomes, including income status and housing insecurity. PWID with IE have high rates of experiencing homelessness, with Canadian retrospective cohort studies reporting rates as high as 17–46% [5,8,9]. Homelessness has been associated with a significantly increased risk of death compared to the age-matched general population [10]. People experiencing homelessness are more likely to have a high burden of acute and chronic diseases and increased disease severity, due to factors including extreme poverty, difficulty accessing health services, and challenges in maintaining treatment engagement [11]. PWID are also less likely to have reliable access to primary care [12].

PWID often experience structural barriers and stigma when accessing acute care, which can deter them from seeking or engaging with health services [13–15]. The use of peer support workers with lived experience in hospitals has been proposed as a destigmatizing intervention [16]. Use of peer workers for hospitalized people who use drugs (PWUD) has yet to be widely implemented, though has been shown to increase treatment engagement, building trust with healthcare providers, assisting with discharge planning, and acting as a bridge between hospital and community settings [17,18]. The role of peer workers in supporting PWID with IE specifically has not yet been explored.

The adverse outcomes and higher long-term mortality rates among PWID with IE have spurred calls for targeted efforts to address the unique needs of PWID after IE [19,20]. The use of multidisciplinary endocarditis teams has been recommended as an intervention to improve health outcomes after IE [21,22], and have typically included medical and surgical specialties involved in the acute care of IE, such as cardiology, cardiac surgery, microbiology, and infectious diseases. However, the concept of the endocarditis team has yet to be adapted to include a multidisciplinary team with expertise in both the medical and social complexities of PWID with IE [20]. The Second Heart Program is a novel clinical program intended to address this gap. This study will assess the feasibility and acceptability of a multidisciplinary team designed to address the unique needs of PWID with IE, and to transition with them from hospital to community.

## Methods and analysis

### Study design

A convergent mixed-methods study design will be used to test the feasibility of this intervention [23]. We will concurrently collect quantitative and qualitative data and ‘mix’ at the interpretation stage of the study to answer our research questions. It is a single group study (i.e., non-randomized). We report this study protocol in accordance with the SPIRIT guidelines [24] (see S1 Checklist for the checklist, see S1 Fig for the SPIRIT Figure) and TIDier checklist [25] (see S2 Checklist).

### Study settings and eligibility criteria

Recruitment for the study will take place at two academic tertiary acute care hospitals in Hamilton, Ontario–St. Joseph’s Hospital and the Hamilton General Hospital. Adult patients (age 18 years and older) residing in Hamilton with a diagnosis of IE and a history of injection drug use within 3 months of time of enrolment will be eligible for the study. Patients will be ineligible if they do not reside in the City of Hamilton or do not understand English. The study research coordinator will enroll participants and obtain consent. All participants will provide verbal informed consent due to COVID-19 pandemic restrictions limiting in-person interaction between participants and researchers. A copy of the consent form is available upon request to the corresponding author.

### Sample size

Using local data from 2008–2017, 86 PWID had surgically-managed IE, with 42 surgically-managed cases in 2016–2017 alone. This data excludes the larger number of IE cases that are managed medically. We estimate a total volume of IE cases amongst PWID as 40–50 per year locally. Our target sample size is 40 intervention participants, which is sufficient to assess the feasibility of our intervention but will not be powered to assess statistical significance of patient outcomes. We expect that we will successfully enroll 50% of patients approached. This sample size can establish that the rate of success for enrolment will be between 39% and 61% with 95% confidence.

### Intervention

The Second Heart Program connects PWID with IE with a multidisciplinary team designed to address their medical and social needs. This team, known as “The Second Heart Team”, will support participants during hospitalization and for a period of one-year after discharge into the community. The six components of the study intervention are: (1) peer support worker with lived experience, (2) systems navigator, (3) addiction medicine physician, (4) primary care physician, (5) infectious diseases specialist, and (6) cardiovascular surgeon (if indicated). Each component is described below.

**(1) Peer support worker.** The peer support worker will provide harm reduction education and informal support to the participants while in hospital. Upon transition to community, peer support workers will continue to provide individualized support to participants, which may include connecting them to harm reduction or treatment services, providing informal counseling or social support, and assisting in attending medical appointments. The peer support worker will first make contact with the participant in hospital, then weekly in the first month post-discharge, and every two weeks thereafter, or more frequently upon participant request.
  In this study, peer support workers are trained and employed by the Canadian Mental Health Association in Hamilton, Ontario, and are seconded to support the participants of the Second Heart Program on a part-time basis.
**(2) Systems navigator.** A systems navigator has expertise in assisting clients with accessing and engaging with complex health and social systems. In this study, the systems navigator will assist participants in accessing medical services, community resources, housing, income supports, transportation, and other social services. The systems navigator will first make contact with the participant in hospital for an intake assessment identifying the participant’s needs and priorities and to develop an individualized care plan. After discharge, the systems navigator will reach out to participants every two weeks for the first 3 months, and monthly thereafter, or more frequently upon participant request.
  In this study, the systems navigator is a registered social worker with extensive experience working with people who use drugs. They are primarily employed by an urban academic tertiary care hospital and seconded to support the participants of the Second Heart Program on a part-time basis.
**(3) Addiction medicine physician.** Participants will be offered consultation from a physician with the Inpatient Addiction Medicine Service while in hospital. The physician may offer medication-assisted treatment if indicated. If treatment is initiated, a transfer of care will be arranged to an outpatient addiction medicine provider upon discharge. If participants decline addiction medicine consultation while in hospital, they can opt to be referred to an outpatient addition medicine provider at any time during the course of the study.
**(4) Primary care physician.** If a patient does not have a primary care physician at time of admission, they will be connected to one prior to discharge. Selection of a primary care physician will be individualized for each participant, with consideration given for preferred practice model (shelter-based clinic, academic or community-based family health team, or community health centre) and geographic accessibility. Efforts will be made to ensure the participant has an appointment with their designated primary care physician within two weeks of discharge from hospital.
**(5) Infectious diseases specialist.** All participants will be referred to a designated infectious diseases physician who is associated with the Second Heart Program and can provide low-barrier access to care via a walk-in model at a local harm reduction agency or the hospital-based outpatient clinic. This infectious disease physician will provide follow-up in accordance with the standard of care.
**(6) Cardiovascular surgeon.** If participants require cardiovascular surgery follow-up after hospitalization, they will be referred to a designated cardiovascular surgeon associated with the Second Heart Program. The cardiovascular surgeon will provide follow-up in accordance with the standard of care.

Prior to discharge from hospital, a case conference will be arranged including the patient, the in-hospital care team (infectious diseases, internal medicine, cardiology, cardiovascular surgery, social work), and the Second Heart Team as described above. The purpose of the care conference is to review the discharge plan and address any anticipated barriers to a safe discharge and successful transition to the community.

Participants will also be provided with a cell phone with a prepaid talk and text plan in order to facilitate contact with the Second Heart Team for the duration of the study.

There are no limitations in terms of other care or supports (medical or non-medical) participants are eligible to access while enrolled in the study intervention.

### Outcomes

The overall objective of this study is to assess the feasibility of the Second Heart Program. For the following research questions, the category of consideration based on recommendations for feasibility studies [26] is noted in parentheses.

1. What is the enrollment, completion and drop-out rate of participants? (process)
2. What are the reasons for drop-out among participants? (process)
3. What is the perceived acceptability of the process of the intervention to eligible participants? (process)
4. What is the number and nature of unintended (or negative) outcomes? (process)
5. How often do peer support workers and systems navigator connect with participants? (resources)
6. What is the nature of the supports/contact points provided by peer support workers and system navigator? (resources)
7. What are the program costs (i.e., cell phones, human resources, travel)? (resources)
8. What is the number and nature of challenges in the collection of data throughout study? (management)
9. What is the reinfection, readmission, and re-intervention rates for participants? (scientific)
10. What is the nature of self-reported substance use and use of harm reduction strategies across the intervention? (scientific)
11. What is the mortality rate 1-year post-hospitalization for IE? (scientific)
12. What is the number of touch points with cardiovascular surgery, cardiology, infectious disease, systems navigator, primary care physician, addictions services in hospital and 1-year post-discharge? (scientific)
13. What are the perceived strengths (impacts), weaknesses (challenges), opportunities, and threats of the program from the perspective of patients, peer support workers, healthcare providers?

Success of the project will be measured by the enrollment rate (N = 40) (50%) and retention rate (>75%) in the proposed intervention.

### Data collection

See S1 and S2 Tables for detailed outcome measures, data sources, and timing of data collection, and analysis. Electronic data will be stored in an electronic data capture tool, Research Electronic Data Capture (REDCap) [27,28] hosted by the Department of Family Medicine at McMaster University.

### Data analysis

Means and standard deviations will be used for continuous data and counts and proportions for categorical data. An inductive and deductive theoretical thematic analysis [29] will be completed by the study team, grounded in SWOT (strengths, weaknesses, opportunities, threats) [30]. Rigor will be fostered using recommendations for trustworthiness and authenticity [31]. Mixed analysis will involve examining instances of concordance and discordance in the quantitative and qualitative data [23]. We have no planned statistical, interim, or sub-analyses.

#### Ethics

The study protocol and trial documents have been approved by the Hamilton Integrated Research Ethics Board (Project #7012, current protocol version date October 29, 2020). Substantial amendments will require ethical review. Participant will have the choice to withdraw for any reason from the study at any time during participant by letting any study team member know. There will be no data monitoring committee but rather, conduct of the study will be continuously monitored by the lead investigator and research coordinator via bi-weekly meetings, and through regular meetings with all research team members. Any adverse events because of the intervention will be reported to the research ethics board as per standard procedures.

#### Study status

The study has been actively enrolling participants since January 2021. As of time of submission (July 2021), participant enrolment remains open and data collection is in process.

#### Dissemination

The results of the study will be submitted for peer review for publication in a scientific journal and will be presented at national and international conferences. Authorship will be determined based on ICJME guidelines.

#### Patient and public involvement

In the early stages of project development, a local harm reduction and advocacy group, Keeping Six, was consulted about the Second Heart Program and the planned intervention. Keeping Six is founded and organized by people with lived experience of drug use and those who love and care for them. This group provided key stakeholder feedback for this research study.

In a precursor study, six patients with IDU-IE participated in qualitative interviews about the role of peer workers in supporting them during and after hospitalization [17]. The themes from their interviews greatly informed the role of the peer support worker in this study.

The perspectives of patients as stakeholders in the development of this program will be included through the qualitative interviews that will take place at the 3-month and 12-month milestones. The interviews will elicit feedback directly from patients on their experience of the intervention and what should be considered as the program is expanded.

## Discussion

This protocol describes a pilot study to assess the feasibility and acceptability of a multidisciplinary endocarditis team for people who use drugs. The Second Heart Program will be, to our knowledge, the first multidisciplinary endocarditis team designed specifically for PWID. By transitioning with the participants from hospital to community, the Second Heart Team will provide a continuity of care infrequently offered to PWID after IE.

A limitation of our study is the lack of funding to support the use of provincial administrative databases, thereby limiting participant healthcare utilization data gathered to that of regional hospital databases. If this intervention is found to be feasible, we will aim to conduct a larger trial to evaluate the impact of the Second Heart Program on patient outcomes, particularly re-admission, re-infection, and mortality rates after infective endocarditis.

## Supporting information

S1 Checklist SPIRIT checklist. (DOC)

S2 Checklist TIDier checklist. (DOCX)

S1 Fig SPIRIT figure. (DOC)

S1 Table Data collection map. (DOCX)

S2 Table Participant-related data collection schedule by participant. (DOCX)
