# Supplementary material for: The Second Heart Program—A multidisciplinary team supporting people who inject drugs with infective endocarditis: Protocol of a feasibility study
Source: PLoS One. 2021 Oct 28;16(10):e0256839. doi: 10.1371/journal.pone.0256839 (PMC8553071; doi:10.1371/journal.pone.0256839)
Supplement: S1 Fig — (DOC) [file pone.0256839.s003.doc]

S1 Figure: SPIRIT Study timeline and data collection time points.

|  | ***STUDY PERIOD*** | | | | | |
| --- | --- | --- | --- | --- | --- | --- |
| **TIMEPOINT** | ***-t_1_*** | ***T0***  ***In hospital (baseline)*** | ***T_1_***  ***Month 1*** | ***T_3_***  ***month 3*** | ***T_6_***  ***Month 6*** | ***T_12_***  ***Month 12*** |
| **ENROLLMENT:**  Eligibility screen | X |  |  |  |  |  |
| Informed consent | X |  |  |  |  |  |
| Confirm eligibility | X |  |  |  |  |  |
| INTERVENTION |  |  |  |  |  |  |
| **Assessments:** |  |  |  |  |  |  |
| *Demographic information* |  | X |  |  |  |  |
| *Self-reported substance use, use of harm reduction strategies, housing and income status* |  | X | X | X | X | X |
| *Perceived suitability and acceptability* |  | X |  |  |  | X* |
| *Reinfection* |  |  | X | X | X | X |
| *Readmission* |  |  | X | X | X | X |
| *Reintervention* |  |  | X | X | X | X |
| *Visits with Cardiovascular surgery* |  |  | X | X | X | X |
| *Visits with infectious disease team* |  | X | X | X | X | X |
| *Visits with system navigator* |  | X | X | X | X | X |
| *Visits with primary care physician* |  | X | X | X | X | X |
| *Visits with addictions services* |  | X | X | X | X | X |
| *Visits with peer support worker* |  | X | X | X | X | X |
| *SWOT* |  |  |  | X |  | X* |
| *Mortality* |  |  |  |  |  | X |

Note. “Visits”= any touch point with the named service (in=person, phone, virtual, etc); EMR = electronic medical record. *indicates assessments for peer support workers, healthcare providers, system navigators, other stakeholders.
