# Supplementary material for: The Second Heart Program—A multidisciplinary team supporting people who inject drugs with infective endocarditis: Protocol of a feasibility study
Source: PLoS One. 2021 Oct 28;16(10):e0256839. doi: 10.1371/journal.pone.0256839 (PMC8553071; doi:10.1371/journal.pone.0256839)
Supplement: S1 Table — (DOCX) [file pone.0256839.s004.docx]

S1 Table : Data Collection Map

| **Research outcome** | **Source(s)** | **Timing** | **Analysis** |
| --- | --- | --- | --- |
| Enrollment, completion and drop-out rate of participants | Program records | Post-study | Descriptive statistics |
| Reasons for drop-out | Program records | Post-study | Content analysis |
| Perceptions of suitability and acceptability of intervention process | Participant survey | In hospital, Post-study | Descriptive analysis |
| Number and nature of unintended (or negative) outcomes | Program records | Post-study | Frequency and content analysis |
| Frequency of peer support worker and systems navigator contact with participants | Program records | Post-study | Frequency |
| Nature of the supports/contact points provided by peer support worker and systems navigator | Program records | Post-study | Content analysis |
| Program costs (i.e., cell phones, human resources, travel) | Financial reports | Post-study | Total by expense item |
| Challenges in the collection of data in study | Memo note | Post-study | Content analysis |
| Reinfection, readmission, and reintervention rates | Electronic medical records at Hamilton General Hospital and St. Joseph’s Healthcare Hamilton*, patient survey designed for the study | 1, 3, 6, 12 months post-discharge | Descriptive statistics |
| Nature of self-reported substance use and use of harm reduction strategies across the intervention | Participant survey | In-hospital, 1, 3, 6, 12 months post-discharge | Descriptive statistics |
| Mortality rate 1-year post-hospitalization | Electronic medical records at Hamilton General Hospital and St. Joseph’s Healthcare Hamilton* | 12 months | Descriptive statistics |
| Number of touch points with cardiovascular surgery, cardiology, infectious disease, systems navigator, primary care physician, addictions services in hospital and 1-year post-discharge | Electronic medical records at Hamilton General Hospital and St. Joseph’s Healthcare Hamilton*, patient survey designed for the study  Self-report survey | In hospital, 12-months  1, 3, 6, 12 months post-discharge | Descriptive statistics |
| Perceived strengths (impacts), weaknesses (challenges), opportunities, and threats of the program | One-on-one interviews with patients, peer support workers, addiction medicine physicians, systems navigator, primary care physician, peer support worker coordinator  Open-ended survey for ‘other care providers’ (cardiovascular surgery, internal medicine, cardiology) and community partners | 3 and 12-months for patients; 12-months for other participants | Thematic analysis, grounded in SWOT analysis [18] |

Note. *data collection planned for people who withdraw from study; SWOT=strengths, weaknesses, opportunities, threats.
