# Supplementary material for: The Second Heart Program—A multidisciplinary team supporting people who inject drugs with infective endocarditis: Protocol of a feasibility study
Source: PLoS One. 2021 Oct 28;16(10):e0256839. doi: 10.1371/journal.pone.0256839 (PMC8553071; doi:10.1371/journal.pone.0256839)
Supplement: S2 Table — (DOCX) [file pone.0256839.s005.docx]

S2 Table: Participant-related data collection schedule by participant

| **Study participant** | **In hospital (baseline)** | **Post-discharge** | | | |
| --- | --- | --- | --- | --- | --- |
|  |  | **1-month** | **3-month** | **6-month** | **12-month** |
| Patient | Patient information (EMR and self-report)  Perceived suitability & acceptability questions  Self-reported substance use, harm reduction strategy use  Number of touch points with cardiovascular surgery, cardiology, infectious disease, systems navigator, primary care physician, addictions services, peer support worker (EMR and self-report survey) | Reinfection rate, readmission rate, reintervention rate, self-reported substance use, harm reduction strategy use,  number of touch points with cardiovascular surgery, cardiology, infectious disease, systems navigator, primary care physician, addictions services, peer support worker (EMR* and self-report survey) | Interview (SWOT questions)  Reinfection rate, readmission rate, reintervention rate, self-reported substance use, harm reduction strategy use, number of touch points with cardiovascular surgery, cardiology, infectious disease, systems navigator, primary care physician, addictions services, peer support worker (EMR* and self-report survey) | Reinfection rate, readmission rate, reintervention rate, self-reported substance use, harm reduction strategy use, number of touch points with cardiovascular surgery, cardiology, infectious disease, systems navigator, primary care physician, addictions services, peer support worker (EMR* and self-report survey) | Patient information (EMR and self-report)  Interview (SWOT questions) + Perceived acceptability questions  Mortality rate  Reinfection rate, readmission rate, reintervention rate, self-reported substance use, harm reduction strategy use, number of touch points with cardiovascular surgery, cardiology, infectious disease, systems navigator, primary care physician, addictions services, peer support worker (EMR* and self-report survey) |
| Peer support worker |  |  |  |  | Interview (SWOT questions) + Perceived acceptability questions |
| Addiction medicine physician |  |  |  |  | Interview (SWOT questions) + Perceived acceptability questions |
| System navigator |  |  |  |  | Interview (SWOT questions) + Perceived acceptability questions |
| Primary care physician |  |  |  |  | Interview (SWOT questions) + Perceived acceptability questions |
| Peer support worker coordinator |  |  |  |  | Interview (SWOT questions) + Perceived acceptability questions |
| Clinician who co-manage patients |  |  |  |  | Open-ended survey (SWOT questions) +  Perceived acceptability questions |
| Other community partners |  |  |  |  | Open-ended survey (SWOT questions) +  Perceived acceptability questions |

Note. *data collection planned for people who withdraw from study; EMR=Electronic medical record; SWOT=Strengths, weaknesses, opportunities
